# Vertebral Artery Caught in the Fracture Gap after Traumatic C2/3 Spondylolisthesis

**DOI:** 10.1155/2017/9179647

**Published:** 2017-08-01

**Authors:** Ralf Henkelmann, Christoph Josten, Stefan Glasmacher, Christoph-Eckhard Heyde, Ulrich Josef Albert Spiegl

**Affiliations:** University of Leipzig, Clinic and Polyclinic of Orthopedic, Trauma and Plastic Surgery, Liebigstrasse 20, 04103 Leipzig, Germany

## Abstract

**Background Context:**

Patient with a C2 fracture and entrapment of the right vertebral artery in the fracture gap.

**Purpose:**

Presentation of a case with follow-up until end of treatment.

**Study Design:**

Case report.

**Methods:**

A 25-year-old woman was brought into our emergency room after falling while riding a horse. She complained of pain in the cervical spine. Clinical examinations showed* local tenderness* at the upper cervical spine and painful impairment of the mobility of the neck, with no signs of neurological impairment. Radiological diagnostics revealed a traumatic C2/3 spondylolisthesis. A computer tomography (CT) angiographic scan showed a dislocation of the right vertebral artery into the fracture gap without injury to the artery. Open reduction and osteosynthesis were considered of too high risk. Therefore, we conducted fracture treatment with closed reduction and halo fixation. After removal of the halo fixator, the patient was given a soft cervical collar and was advised to rest for additional 6 weeks before beginning gradual activity.

**Results:**

Conventional follow-up revealed osseous consolidation and a CT angiographic scan showed consistent blood flow to the artery.

**Conclusion:**

Halo fixation was a safe and effective therapy strategy in the case of vertebral artery entrapment after traumatic C2 spondylolisthesis.

## 1. Background 

Vertebral artery injury (VAI) is mainly associated with traumatic cervical spine injuries, particularly in cases involving fractures. It is estimated to occur in approximately 0.5% of cases involving blunt trauma. This rate increases to 20% in cases of cervical spine fractures. One of the risk factors for VAI is fracture displacement in the transverse foramen of 1 mm or more [1]. Previous reports associate VAI with high cervical fractures (C1–C3), particularly in C2 fractures which affect the foramen transversarium. Effendi et al. classified fractures of the ring of the axis and proposed treatment strategies, which were modified by Levine and Edwards as well as Josten [2–4]. Common dissection of the vertebral artery is seen in 0.01% of cases with head or neck trauma and vertebral fracture is a significant risk factor for a vertebral dissection. Also, the rate of complications, such as cerebral ischemia, is increased [5]. Artery dissections are responsible for around 20% of strokes in young patients [6]. However, to our knowledge, no report is published detailing treatment of a vertebral artery caught in the fracture gap after a traumatic C2/3 spondylolisthesis [7].

## 2. Presentation of the Case 

A 25-year-old woman fell while riding a horse. She was brought into our emergency department and complained of immediate and intense cervical pain without neurological deficit. She had no previous medical history.

**Figure 1:**
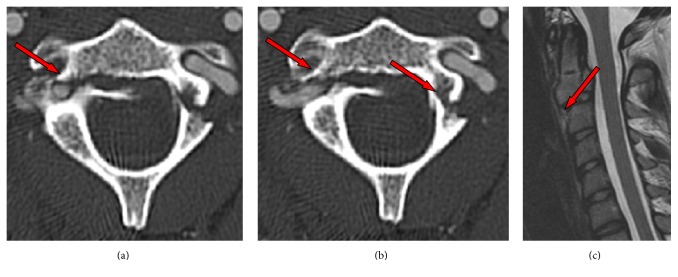
((a) and (b)) Primary CT with contrasted dislocation of the artery in the fracture gap; (c) sagittal MRI arrows point to a small hematoma under the anterior longitudinal ligament.

Upon initial clinical examination, the patient had isolated intense cervical neck pain while wearing a hard cervical collar. She was fully oriented and had no motor paralysis or sensory disturbance. She complained of nausea that had begun right after receiving intravenous pain medication, which then subsided spontaneously. There was no sign of any head injury or concussion. A computer tomography scan revealed a traumatic C2/3 spondylolisthesis type Effendi II and Levine and Edwards type II (Figure 1). Magnetic resonance tomography did not show any lesion of the anterior longitude ligament. Because of the fracture involvement with the transverse foramen, an additional computer tomography with contrast media was performed to rule out VAI. This image showed a dislocation of the right vertebral artery into the fracture gap (Figure 1).

## 3. Treatment Strategy and Follow-Up

**Figure 2:**
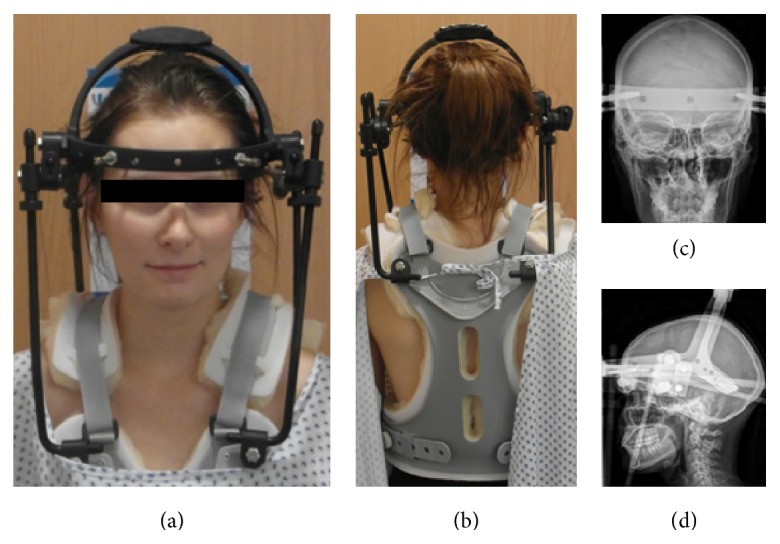
Halo fixation. (a) Front view, (b) rear view, (c) frontal view, and (d) lateral view.

We opted for a procedure with a closed reduction and stabilization with use of a halo fixator (Figure 2). The procedure was performed while the patient was awake with intravenous analgesia in the operating theater under surgery anesthetic stand-by. Hereby, the neurology was continuously controlled and the reduction was done very gently by axial traction. After closed reduction and halo fixation, the neurology was controlled intensively. Postoperatively, the pain decreased considerably and the neurological status was uneventful at any time during the next three days. Therefore, we abstained from further vascular imaging.

**Figure 3:**
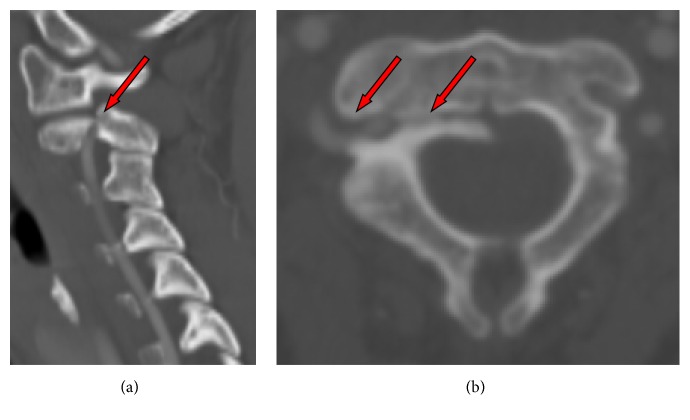
CT with contrast of fracture featuring a partial ossification and artery dislocation after three months: (a) sagittal view and (b) axial view.

**Figure 4:**
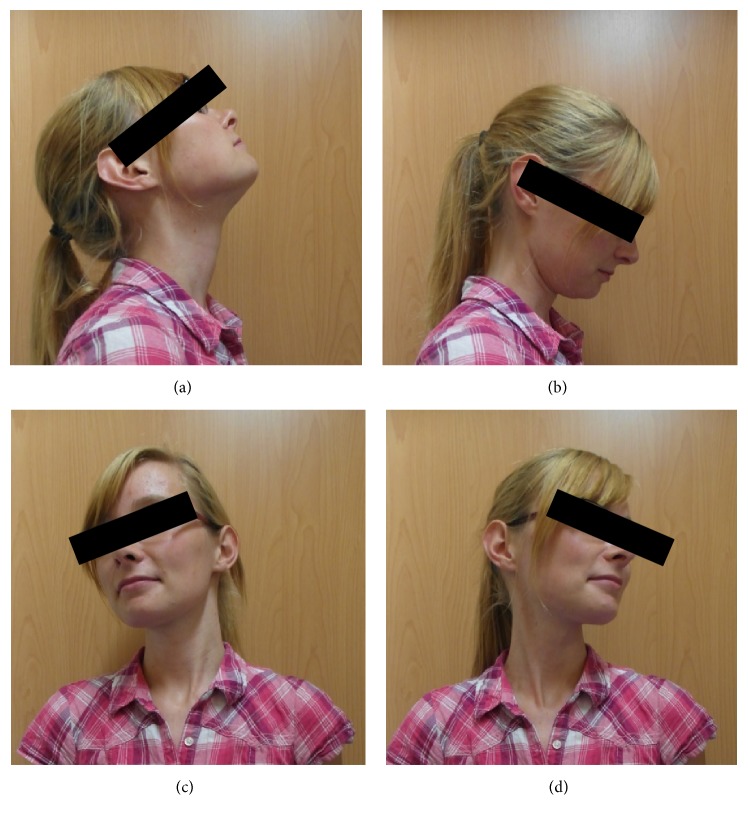
Range of motion after 5.5 months (1.5 months after removal of halo fixation): (a) declination, (b) inclination, and (c) and (d) right and left rotation.

Radiological examination performed two and six weeks postoperatively showed no loss of reduction. Clinical examinations in regular intervals in our outpatient clinic showed no local infection of the halo fixation pins and continuous pain reduction. After 3 months, a CT scan with contrast was performed, showing only partial ossification (Figure 3). The halo fixator was left in situ for another month. Four months after trauma, the radiograph examination demonstrated blurring of the fracture gap, consistent with osseous consolidation and correct upper cervical alignment. Thus, the halo fixator was removed and a soft cervical collar was fitted. At this point, intensive physiotherapy with isometric training to improve muscle strength and cervical spine motion began. After six weeks, functional radiographs were taken, showing a stable osseous consolidation and normal good cervical spine function. Use of the soft collar was discontinued. The fixation pins healed without event and the patient did not complain of any cervical pain. Her range of motion was 60° movement to the right and left and featured a slightly restricted inclination and declination (Figure 4). No neurologic deficit was visible. Furthermore, the patient was free of symptoms after removal of the soft collar.

## 4. Discussion 

This case report describes the rare case of a vertebral artery caught in the fracture gap after a traumatic C2/3 spondylolisthesis (Effendi II, Levine and Edwards II, and Josten III) with intact anterior ligament treated during halo fixation [2–4]. The treatment resulted in a good clinical outcome without neurologic deficit. Another option is an open approach with screw osteosynthesis of the vertebral C2 arch. There would be a risk of damaging the involved left vertebral artery in addition to the high risk of dissecting the right vertebral artery, which was caught in the fracture gap and therefore hardly visible perioperative.

A systematic review by Li et al. concluded that the majority of C2/C3 fractures could be treated with external immobilization. The indication for a surgical treatment remains controversial [8]. Levine and Edwards recommend a posterior stabilization for type II, IIa, and III fractures depending on the condition of the disc space and posterior longitudinal ligament [4]. An anterior fixation is beside the posterior stabilization a possible operative alternative and is mainly recommended for instable fractures (Effendi III, Josten IV) [9]. As a result of the unremarkable C2/3 intervertebral disc, no ventral approach was considered. Several studies showed good results with different strategies of conservative treatment with halo fixation, a cervical collar, or a Minerva cast [10–12]. ElMiligui et al. described contrary results with nonunion, instability, or persistent pain [13]. Longo et al. concluded in their review that the most important, but rare, complication is nonunion [14]. Due to the fact that posterior stabilization can also be the cause of nonunion, the indication is limited [15].

A high complication rate for pin loosening, pin infection, halo ring migration, nerve injury, and cranial or dural penetration with halo fixation is still reported. Pin tract infection is the most common complication. In most of the cases, aseptic loosening or focal infection does not require the pin to be removed; however, in some cases, local debridement and pin removal are necessary [16].

As Strohm et al. concluded, the halo fixator has some indications to consider alternatives, particularly in terms of biomechanical stability [12]. One has to consider that a higher morbidity and mortality are associated with older people. In this case, a young woman was treated with halo fixation [17].

## 5. Limitation

We tried to compute CT-3D-reconstructions for a better display of the fracture pattern but due to the orientation of the fracture lines and the artery it was not possible to picture it better than with axial CT images. The patient moved away whereby no longer follow-up was possible.

## 6. Conclusion

In our opinion, in this rare case featuring an intact anterior ligament and no neurological deficit, a halo fixation is an adequate treatment option.

## Abbreviations

VAI: Vertebral artery injury
CT: Computer tomography
C: Cervical vertebral body.
